# Objectively-assessed outcome measures: a translation and cross-cultural adaptation procedure applied to the Chedoke McMaster Arm and Hand Activity Inventory (CAHAI)

**DOI:** 10.1186/1471-2288-10-106

**Published:** 2010-11-29

**Authors:** Corina Schuster, Sabine Hahn, Thierry Ettlin

**Affiliations:** 1 Research Department, Reha Rheinfelden, Salinenstrasse 98, 4310 Rheinfelden, Switzerland; 2 Occupational Department, Reha Rheinfelden Salinenstrasse 98, 4310 Rheinfelden, Switzerland; 3 Department of Neurology, Medical faculty, University of Basel, Klingelbergstrasse 61, 4056 Basel, Switzerland

## Abstract

**Background:**

Standardised translation and cross-cultural adaptation (TCCA) procedures are vital to describe language translation, cultural adaptation, and to evaluate quality factors of transformed outcome measures. No TCCA procedure for objectively-assessed outcome (OAO) measures exists. Furthermore, no official German version of the Canadian Chedoke Arm and Hand Activity Inventory (CAHAI) is available.

**Methods:**

An eight-step for TCCA procedure for OAO was developed (TCCA-OAO) based on the existing TCCA procedure for patient-reported outcomes. The TCCA-OAO procedure was applied to develop a German version of the CAHAI (CAHAI-G). Inter-rater reliability of the CAHAI-G was determined through video rating of CAHAI-G. Validity evaluation of the CAHAI-G was assessed using the Chedoke-McMaster Stroke Assessment (CMSA). All ratings were performed by trained, independent raters. In a cross-sectional study, patients were tested within 31 hours after the initial CAHAI-G scoring, for their motor function level using the subscales for arm and hand of the CMSA. Inpatients and outpatients of the occupational therapy department who experienced a cerebrovascular accident or an intracerebral haemorrhage were included.

**Results:**

Performance of 23 patients (mean age 69.4, SD 12.9; six females; mean time since stroke onset: 1.5 years, SD 2.5 years) have been assessed. A high inter-rater reliability was calculated with ICCs for 4 CAHAI-G versions (13, 9, 8, 7 items) ranging between r = 0.96 and r = 0.99 (p < 0.001). Correlation between the CAHAI-G and CMSA subscales for hand and arm was r = 0.74 (p < 0.001) and r = 0.67 (p < 0.001) respectively. Internal consistency of the CAHAI-G for all four versions ranged between α = 0.974 and α = 0.979.

**Conclusions:**

The TCCA-OAO procedure was validated regarding its feasibility and applicability for objectively-assessed outcome measures. The resulting German CAHAI can be used as a valid and reliable assessment for bilateral upper limb performance in ADL in patients after stroke.

## Background

Language is a historically developed form of human communication that reflects culture, traditions, and customs of a country and its inhabitants. It is specific for each country and contains words, symbols and characters or syntax that have a unique meaning for its users [1]. Thus, standardised translation and cross-cultural adaptation (TCCA) procedures are vital to describe language translation, cultural adaptation, and to evaluate quality factors of transformed outcome measures. For example, 'Please dial 144.' is one of the activities that will be assessed with the German version of Chedoke McMaster Arm and Hand Activity Inventory (CAHAI-G). Modifying the telephone number is a cultural adaptation from the original Canadian English CAHAI developed by Barreca and colleagues in 2004 [2].

In 2009, PubMed has indexed about 22 articles describing a TCCA procedure for patient-reported outcome measures (PRO). Different TCCA-PRO procedures have been developed by research groups [3-6], in particular to translate and adapt assessments in several other languages and implement international multicentre studies. Acquadro and colleagues carried out an extensive literature review and found 17 TCCA-PRO guidelines, and 22 articles that reviewed TCCA-PRO requirements and strategies, and gave recommendations for TCCA-PRO procedures [7]. Nevertheless, those investigations focused on PRO-based assessments, which are subjectively reported by patients. Assessments for objective scoring of assessors were not specifically addressed.

Whereas the development of TCCA-PRO requires an understanding of the general population, objectively-assessed outcome measures (TCCA-OAO) require knowledge of profession-specific vocabulary. The main focus for TCCA-OAO is to explicitly describe the task to be performed by patients as well as the objective scoring instructions for assessors. To our knowledge, no official TCCA guideline exists that specifically addresses OAOs. It is nevertheless essential to apply a standardised procedure for these assessments to 1) carry out translations, 2) adapt test instructions to region-specific and profession-specific vocabulary, 3) review and test the translation for comprehensiveness and practicability by professionals themselves, and 4) evaluate quality factors for its use in a foreign country. These goals have been addressed for TCCA-OAO in this study by adapting a multistep procedure that was recommended by Acquadro et al. [7].

The aim of this study was to develop and evaluate a procedure for TCCA-OAOs that can provide a generic guidance and standard process for OAOs. The proposed procedure was evaluated by TCCA of the English CAHAI to a general German-speaking audience regarding inter-rater reliability and convergent construct validity of the German version.

Regaining arm and hand function is one of the most frequently named aims for patients after stroke [2].To this end, OAO measures are essential to set appropriate rehabilitation goals, to record the patient's treatment course, and to evaluate service delivery. OAOs are also important to compare health status among different nations, for international health campaigns, and development of treatment options. Barreca and colleagues developed the CAHAI focussing on upper limb function assessment in patients after stroke. The CAHAI items are based on interviews performed with patients having a hemiparesis as well as their caregivers. In total 751 items have been generated, which finally resulted in 13 bilateral ADL-relevant tasks [2]. Quality factors of CAHAI have been investigated showing a high inter-rater reliability (ICC = 0.98), a minimal detectable change of 6.3 points, and a high correlation with the Action Research Arm Test (ARAT, r = 0.93) and the CMSA (subscale arm and hand r = 0.81) to support convergent validity [8]. Subsequently three shorter versions of CAHAI-13 were developed by the original authors: CAHAI-7, CAHAI-8, and CAHAI-9 [9]. Patients can reach a score for CAHAI 7 ranging between 7 to 49, for CAHAI 8 between 8 to 56, for CAHAI 9 between 9 to 63, and for CAHAI 13 between 13 to 91[8,9].

Using the proposed TCCA procedure, a German version (CAHAI-G) was derived in this study. Subsequently inter-rater reliability and convergent construct validity of the German version was determined in a patient study. Feasibility and applicability of the TCCA procedure was confirmed using these quality factors.

## Methods

A formal TCCA-OAO procedure was developed and implemented for CAHAI. The detailed procedure, implementation, and CAHAI-G validation is described as follows.

### The translation and cross-cultural adaptation procedure (TCCA)

An eight-step procedure for TCCA-OAO was developed based on the TCCA-PRO procedure proposed by Beaton et al. [3] and Acquadro et al.'s review recommendations [7]. Both articles suggested following a generic systematic multi-step procedure including more than one forward translation to account for variability in language interpretation. Compared to Beaton's 6-step procedure, the TCCA-OAO was modified and extended to emphasise importance and time needed to perform standardised high-quality translations. Table 1 summarised all individual steps of TCCA-OAO.

**Table 1:** Eight-step procedure for translation and cross-cultural adaptation of objectively-assessed outcome measures (TCCA-OAO procedure)

Step	Aim	Required personnel
**1**	To produce two independent **forward translations **and make **necessary region-specific adaptations **of the test manual including task and material descriptions, and scoring instructions into the target language.	The two informed^1 ^translators are native speakers of the target language and profession^2^. Translators are aware of the study objectives.

**2**	To **merge **the two forward translations from step 1 to form only one translation. To check comprehensiveness by a therapist/person of the target profession for consistency and adequate vocabulary.	The synthesis is done by another independent and informed person of the target profession or the project manager.

**3**	To **review **layout, grammar, and typography. This can be very time-consuming but it is important to provide an error-free, professional document for all following steps.	This check is done by the project manager or another person not involved in the translation process but with expertise in the target profession.

**4**	To **backward translate **the merged version by an informed person to assure detection of inconsistencies or conceptual errors, and discrepancies [3,7].	The back translator should be bilingual or a native speaker of the source language and should have not seen the original before.

**5**	To **review **all translations, including all photo or video material showing the necessary test-specific material (e.g. wooden cubes, cups, clothes or zippers). The review should verify a consistent translation and adaptation process [3]. If the translation process fails, a second forward and backward translation is recommended [16].	This review of all translations and created documents should be done by the original authors including the material that will by used.

**6**	To **adapt and re-check **the merged forward translation based on the review comments and for grammatical, typographical or other errors, in particular, for consistency in the task and scoring descriptions, and client instructions (quality control step).	The project manager or one of the forward translators could do this check.

**7**	To **pre-test **the translated version with 2 to 4 patients including the comprehensiveness of the test manual, and the task and scoring descriptions. Emerging discrepancies of scoring or interpretation of results shall be discussed. Based on severity of required adaptations go back to steps 5 or 6.	Two professionals should test the pre-final version with patients.

**8**	To **evaluate **the quality factors of the trans-adapted OAO in patient studies. Depending on the OAO types of validity, reliability and responsiveness have to be determined.	This is the most time and human resources consuming part involving: patients, health professional of different disciplines, a project manager, assistants, and a statistician.

^1^An informed translator has expertise of and is working in the target profession.

^2^Beaton et al. suggested one informed and one uninformed translator. However, this study has found that two informed translators are more applicable for TCCA-OAO since the vocabulary of the target profession would be consistently used.

The widely known and accepted back translation approach was assumed in Step 4 as no scientific evidence exists to favour a two-panel approach [10].

The expert committee meeting, suggested by Beaton et al. [3] for TCCA-PRO, was omitted since informed forward translators were considered and an additional comprehensiveness check was performed by a person of the target profession. Moreover, in contrast to Beaton et al., all translations and necessary material was checked by the original contributors before pretesting with patients. With this approach conceptual errors and misleading scoring of the tested motor functions were avoided.

Furthermore, a TCCA project manager should be made responsible for the whole TCCA procedure, to oversee each step, to make sure that all actors work carefully, to document each step and decision, and to stay in contact with the original authors if discrepancies emerge.

### Implementation of the TCCA-OAO procedure

#### Step 1

The English CAHAI was forward translated by two native German speakers with excellent English language skills, who were aware of the study objectives. As suggested by Beaton et al. one of the translators was not a member of the target profession (uninformed). None of the 13 tasks have been modified except task 2. To fulfil task 2, patients are asked to dial a region-specific emergency number.

#### Step 2

Both translations were synthesised and checked for comprehensiveness by a professional of the target health profession.

#### Step 3

The produced test documents were adapted to the clinic's layout style. They were checked for correct grammar and typography by a secretary.

#### Step 4

The merged German version was backward translated by a bilingually grown up medical doctor.

#### Step 5

The merged German version, the backward translated English version, and photographs of all bought and manufactured objects used in the assessment were sent to the original authors for review.

#### Step 6

All CAHAI-G documents were checked for potentially necessary corrections or adaptations by a therapist after receiving permission from the original authors.

#### Step 7

The German version was pre-tested by three occupational therapists with four patients before starting a patient study to determine practical feasibility and applicability.

#### Step 8

A validation study was performed for CAHAI-G to assess the quality factors.

### The CAHAI and CMSA outcome measures and material

The CAHAI contains 13 real-life items scored from one to seven (highest score). Scoring represents the independence of patients to perform stabilisation or manipulation in activities of daily living (ADL) with the affected upper limb. Score one represents total dependence from another person, whereas score seven shows patient's independence without time or safety concerns, necessary splints or devices. After translating CAHAI 13 into CAHAI-G 13 all items required for CAHAI-G 7, 8, and 9 were available. As in the Canadian CAHAI 7, 8, and 9 the German versions (CAHAI-G 7, 8, 9) contain the corresponding items. The officially translated and approved German version (CAHAI-G 13) was used in the patient study. Due to the broad ADL focus of CAHAI, the complete version requires more than 20 household materials, e.g. fork, knife, plate, telephone, glasses, toothbrush. All necessary materials were described in the translated manual regarding their size, weight, shape, and function. The scoring sheet for CAHAI-G (13 items) is presented in the appendix (Additional file 1). All additional scoring sheets (CAHAI-G 7, 8, 9) and the assessment manual can be obtained from the corresponding author.

Additionally, patient's arm and hand motor functions were assessed with the Chedoke-McMaster Stroke Assessment (CMSA) [11,12]. The CMSA evaluates physical impairment and activity level of stroke patients. The impairment subscales (postural control, arm, hand, leg, foot) were measured on a seven-point scale (1 = low, 7 = high) according to seven stages of motor recovery [13]. Additionally, shoulder pain of the affected body side was evaluated on the same seven-point scale using a further subscale. In this study only subscales for arm, hand, and shoulder pain were analysed. The CMSA was shown to be a valid and reliable assessment [11,12]. CMSA requires material only for the highest level of motor function in subscale hand: a tennis ball, a cup, and a can with a volume of 250 ml.

### CAHAI-G validation study (Step 8)

#### Participants and study setting

Inpatients and outpatients of the occupational department were recruited for the study if eligible between May and September 2008. The selection criteria are summarised in Table 2. The cantonal ethics committee of the canton Aargau, Switzerland, approved the study as part of a larger project. After giving written informed consent patient's performance of the CAHAI-G 13 were tested and scored by the second author.

**Table 2:** Patient selection criteria

Inclusion criteria	Exclusion criteria
- Experienced a first-ever cerebrovascular accident, a intracerebal or a subarachnoid haemorrhage	- Patients with no motor function in the affected upper limb
	- Patients with apraxia
- Minimal motor function in the affected upper limb	- Patients with global aphasia
- Older than 18 years	- Patients with a pre-existing limiting injury of the affected and non-affected upper limb
- Written informed consent	- Patients with additional neurological diseases

#### Test evaluation and assessor training

All CAHAI assessors were trained by the second author using video material and the translated manual before starting the pre-testing and the patient study. Four patients previously assessed by the second author were scored during a training video rating by the other two assessors. Those patients were not included in the study. Discrepancies, comments and questions regarding instructions or patient scoring were discussed among all assessors. In total, each assessor of the CAHAI underwent a training of ten to 13 hours.

The assessor of CMSA received a two day intensive training course including fundamentals of CMSA, its practical application, and patient testing. All involved occupational therapists had a six to ten years experience in rehabilitation of patients after a lesion of the central nervous system. All test sessions were videotaped.

#### Test situation and video rating

Patients were tested twice within 31 hours: once with the CAHAI and once with the CMSA. As the CAHAI has to be performed while sitting, except for task 12 and 13, the therapist was sitting next to the patient's affected side to assist if necessary. The task was initially performed and shown by the therapist using hand and arm corresponding to the patient's affected side. After observing the therapist, patients performed each task themselves from a standardised starting position as described in the manual. Two other trained occupational therapists rated videos independently. They were allowed to view the video as often as necessary to score each task.

After the CAHAI evaluation, the patient's arm and hand motor function, and shoulder pain were evaluated by a trained assessor blinded to the CAHA-G scoring. For this purpose, subscales arm, hand, and shoulder pain of the CMSA were used. Patients were given two attempts to perform each task for both assessments.

### Statistical analysis

Descriptive data was calculated representing frequencies, means, and standard deviations for patient's personal data and outcome measures where appropriate. Convergent construct validity was estimated by calculating Spearman's rank correlation coefficient for correlation of the total CAHAI-G score and the CMSA subscales for hand, arm and shoulder pain. Corresponding p-values were derived.

Inter-rater reliability was determined by calculating intraclass correlation coefficients (ICCs) using a two-way random model with absolute agreement and 95% confidence interval (CI) [14]. Values for single measure reliability are provided. Cronbach's alpha was calculated to describe the internal consistency of the CAHAI-G. All statistical analyses were performed with the statistical package for social sciences (SPSS) version 16 for all four versions of CAHAI-G: CAHAI-G-7, CAHAI-G-8, CAHAI-G-9, and CAHAI-G-13. Alpha was set at p ≤ 0.005.

## Results

### TCCA procedure

All eight steps were followed as proposed in the TCCA-OAO procedure. We noticed a difference between both forward translations regarding the used vocabulary. The synthesis step was very important to produce one sound forward translation. Therefore, we recommend informing forward translators about the topic or choosing translators from the target-profession.

Layout adaptations and verifying grammar and typography required more time than expected. Both performed checks were absolutely necessary to clarify understanding and interpretation of tasks and scoring instructions.

### Study population and assessment scoring

During the recruitment phase 23 patients (mean age 69.4, SD 12.9; 6 females) met the selection criteria and were included in the study. Time since the event ranged between 26 days to eight years (mean 1.5 years, SD 2.5 years). In nine of 22 right-handed patients the left body side was affected. Fifteen patients were reliant on a wheelchair, three patients used a stick, one patient used a rollator walker, and four patients did not use any walking devices. Patients mean scores of all three raters for the CAHAI-G version with seven, eight, nine and 13 items, and the corresponding scores for CMSA subscales hand, arm, and shoulder pain are shown in Table 3. For all single CAHAI-G tasks (one to 13) mean scores for all three raters are provided in Table 4.

**Table 3:** Patient's scores for all four versions of the CAHAI-G and the corresponding CMSA subscale scores

Patient	CAHAI-G 7		CAHAI-G 8		CAHAI-G 9		CAHAI-G 13		CMASA	CMSA	CMSA
	
	Mean	SD	Mean	SD	Mean	SD	Mean	SD	Hand	Arm	Shoulder pain
**1**	32.0	3.6	34.3	4.2	36.0	5.3	48.7	5.5	3	5	4

**2**	21.0	3.6	24.0	3.6	26.7	4.7	43.0	6.6	3	4	6

**3**	27.0	5.3	29.7	6.7	30.7	6.7	41.7	10.2	3	3	6

**4**	49.0	0.0	56.0	0.0	63.0	0.0	91.0	0.0	5	5	6

**5**	11.7	2.5	14.0	3.0	15.0	3.0	23.7	3.8	2	2	3

**6**	47.0	2.6	54.0	2.6	61.0	2.6	86.3	4.0	5	4	6

**7**	10.0	1.7	12.7	1.5	14.3	2.1	22.7	4.0	3	4	6

**8**	16.7	4.7	20.0	5.3	22.0	5.3	29.3	6.7	3	4	6

**9**	48.0	1.0	55.0	1.0	61.7	1.2	89.7	1.2	4	5	6

**10**	8.7	0.6	10.7	0.6	11.7	0.6	15.7	0.6	3	2	4

**11**	14.7	2.1	16.7	2.1	18.7	2.1	31.0	2.0	3	3	6

**12**	37.0	2.6	43.3	2.5	48.7	3.1	67.3	3.1	3	5	6

**13**	36.7	6.7	41.3	6.5	46.3	7.1	58.0	6.6	7	6	6

**14**	40.7	2.5	46.0	3.6	50.0	3.6	66.0	4.4	5	6	6

**15**	43.0	3.6	48.3	5.1	54.0	6.1	69.0	6.2	5	6	6

**16**	44.3	2.3	50.0	3.0	56.3	3.1	80.0	6.2	5	5	6

**17**	36.7	0.6	40.7	0.6	45.3	0.6	62.7	2.5	3	5	6

**18**	14.0	1.0	17.3	1.5	21.3	1.5	31.0	2.6	3	3	6

**19**	43.3	1.5	49.0	1.7	51.0	1.0	72.3	3.5	4	4	6

**20**	38.0	3.0	44.0	3.0	49.0	3.6	60.7	4.5	4	5	6

**21**	47.0	2.6	53.3	3.8	60.3	3.8	86.0	5.3	7	7	7

**22**	7.7	1.2	9.0	1.7	10.0	1.7	16.0	3.0	3	2	2

**23**	27.0	1.0	30.0	0.0	31.0	0.0	49.0	1.7	4	3	4

CAHAI-G = Chedoke Arm and Hand Activity Inventory, CMSA = Chedoke-McMaster Stroke Assessment, SD = standard deviation,

**Table 4:** CAHAI-G mean values for all three raters for each CAHAI-G task

Task	Patient		1	2	3	4	5	6	7	8	9	10	11	12	13	14	15	16	17	18	19	20	21	22	23
**1**	**Open jar of coffee**	Mean	5.3	3.0	6.3	7.0	1.0	6.7	1.3	1.7	6.3	1.0	2.0	6.0	5.7	6.0	6.0	6.7	6.7	1.3	7.0	4.3	6.3	1.3	6.0
		
		SD	1.5	0.0	0.6	0.0	0.0	0.6	0.6	0.6	0.6	0.0	1.7	0.0	1.2	0.0	1.0	0.6	0.6	0.6	0.0	0.6	0.6	0.6	0.0

**2**	**Call 144**	Mean	5.3	3.7	3.7	7.0	1.0	7.0	1.0	4.0	7.0	1.0	2.0	5.7	5.0	4.3	6.7	6.0	6.0	3.0	6.7	6.3	6.7	1.0	4.7
		
		SD	1.2	0.6	0.6	0.0	0.0	0.0	0.0	2.0	0.0	0.0	1.0	1.5	2.0	1.5	0.6	0.0	1.0	0.0	0.6	0.6	0.6	0.0	2.3

**3**	**Draw line with ruler**	Mean	3.0	3.0	4.7	7.0	2.0	6.3	1.7	3.0	7.0	2.0	2.0	5.3	5.7	6.3	6.7	5.3	4.0	2.0	5.7	5.0	6.3	1.3	3.7
		
		SD	1.0	1.0	2.1	0.0	0.0	1.2	0.6	1.0	0.0	0.0	0.0	1.2	1.2	0.6	0.6	0.5	0.0	0.0	0.6	1.0	1.2	0.6	0.6

**4**	**Pour glass of water**	Mean	3.7	3.7	3.3	7.0	1.7	7.0	2.3	2.0	7.0	1.3	2.7	6.7	4.3	6.0	5.3	6.3	5.0	2.0	6.0	6.3	7.0	1.0	3.7
		
		SD	0.6	0.6	1.5	0.0	0.6	0.0	0.6	0.0	0.0	0.6	0.6	0.6	1.5	0.0	1.2	0.6	0.0	1.0	0.0	0.6	0.0	0.0	0.6

**5**	**Wring out washcloth**	Mean	6.7	2.7	3.3	7.0	2.3	7.0	1.7	3.0	7.0	1.3	3.0	4.7	4.7	6.7	6.3	6.7	4.0	2.7	6.0	5.7	7.0	1.0	5.0
		
		SD	0.6	1.2	0.6	0.0	0.6	0.0	0.6	1.0	0.0	0.6	0.0	1.2	1.2	0.6	0.6	0.6	0.0	0.6	0.0	0.6	0.0	0.0	1.0

**6**	**Do up five buttons**	Mean	3.3	2.7	4.7	7.0	2.3	6.7	1.0	1.0	7.0	1.0	1.7	5.3	6.3	5.3	7.0	6.7	5.3	1.0	7.0	6.3	6.7	1.0	1.3
		
		SD	0.6	1.2	1.5	0.0	1.2	0.6	0.0	0.0	0.0	0.0	0.6	0.6	0.6	1.2	0.0	0.6	1.2	0.0	0.0	0.6	0.6	0.0	0.6

**7**	**Dry back with towel**	Mean	4.7	2.3	1.0	7.0	1.3	6.3	1.0	2.0	6.7	1.0	1.3	3.3	5.0	6.0	5.0	6.7	5.7	2.0	5.0	4.0	7.0	1.0	2.7
		
		SD	0.6	0.6	0.0	0.0	0.6	0.6	0.0	1.0	0.6	0.0	0.6	1.2	1.0	0.0	1.0	0.6	0.6	1.0	1.0	1.0	0.0	0.0	1.5

**8**	**Put toothpaste on toothbrush**	Mean	2.3	3.0	2.7	7.0	2.3	7.0	2.7	3.3	7.0	2.0	2.0	6.3	4.7	5.3	5.3	5.7	4.0	3.3	5.7	6.0	6.3	1.3	3.0
		
		SD	0.6	0.0	1.5	0.0	0.6	0.0	0.6	0.6	0.0	0.0	0.0	0.6	1.2	1.2	1.5	1.5	0.0	0.6	0.6	0.0	1.2	0.6	1.0

**9**	**Cut medium resistance putty**	Mean	1.7	2.7	1.0	7.0	1.0	7.0	1.7	2.0	6.7	1.0	2.0	5.3	5.0	4.0	5.7	6.3	4.7	4.0	2.0	5.0	7.0	1.0	1.0
		
		SD	1.2	1.2	0.0	0.0	0.0	0.0	0.6	0.0	0.6	0.0	0.0	0.6	1.0	0.0	1.5	0.6	0.6	0.0	1.0	1.0	0.0	0.0	0.0

**10**	**Zip up the zipper**	Mean	3.0	2.7	3.3	7.0	2.3	6.3	1.7	3.7	7.0	1.0	2.7	4.7	5.7	4.3	2.3	6.0	5.3	4.3	5.3	3.7	5.3	1.0	4.0
		
		SD	0.0	1.2	1.2	0.0	0.6	0.6	0.6	2.1	0.0	0.0	0.6	1.2	1.5	1.5	0.6	1.7	1.2	0.6	1.2	1.2	0.6	0.0	1.7

**11**	**Clean a pair of eyeglasses**	Mean	4.0	2.3	5.3	7.0	1.7	6.3	1.7	1.7	7.0	1.0	1.3	4.7	4.0	6.0	6.0	5.3	3.3	3.3	5.0	6.0	6.3	1.0	1.7
		
		SD	0.0	0.6	2.1	0.0	0.6	1.2	0.6	0.6	0.0	0.0	0.6	1.5	1.0	0.0	0.0	1.5	0.6	0.6	1.0	0.0	1.2	0.0	0.6

**12**	**Place container on table**	Mean	3.7	5.3	1.3	7.0	3.7	6.7	2.0	1.0	7.0	1.0	4.7	4.3	1.0	2.3	2.7	6.3	4.3	1.0	5.0	1.0	7.0	1.0	6.0
		
		SD	0.6	1.2	0.6	0.0	0.6	0.6	1.0	0.0	0.0	0.0	1.5	0.6	0.0	0.6	0.6	0.6	0.6	0.0	1.0	0.0	0.0	0.0	1.0

**13**	**Carry the bag up the stairs**	Mean	2.0	6.0	1.0	7.0	1.0	6.0	3.0	1.0	7.0	1.0	3.7	5.0	1.0	3.3	4.0	6.0	4.3	1.0	6.0	1.0	7.0	3.0	6.3
		
		SD	0.0	0.0	0.0	0.0	0.0	0.0	1.0	0.0	0.0	0.0	1.5	1.0	0.0	1.2	0.0	0.0	0.6	0.0	0.0	0.0	0.0	1.7	0.6

	**Total of means**		**48.7**	**43.0**	**41.7**	**91.0**	**23.7**	**86.3**	**22.7**	**29.3**	**89.7**	**15.7**	**31.0**	**67.3**	**58.0**	**66.0**	**69.0**	**80.0**	**62.7**	**31.0**	**72.3**	**60.7**	**86.0**	**16.0**	**49.0**

SD = standard deviation

### CAHAI-G implementation

All CAHAI-G raters used the whole range of the scoring scale ranging from one to seven to evaluate bilateral arm function in all 23 patients. The most agreements were reached for task 13 ('carry the bag up the stairs'), and the least for task 10 ('zip up the zipper'). Duration of assessment implementation/performance ranged between ten minutes for the CAHAI-G 7 to 35 minutes for the CAHAI-G 13. In 18 out of 23 patients the scoring varied between one to three points for all 13 tasks and among all three raters. In five out of 18 patients both video ratings reached the same scoring but deviated from the direct rating during patient performance in all tasks. The highest congruence among all raters was found in patients achieving a scoring between 13 to 40 points and between 70 to 91 points. The range between 40 to 70 points showed the most variability

Evaluated motor functions with CMSA ranged from two to seven in all patients for subscale hand and shoulder pain. Scores for subscale arm motor function have been evaluated at level two, three, four, five, and seven.

### Reliability

Internal consistency, ICCs, and correlations for all four CAHAI-G versions are shown in Table 5.

**Table 5:** Internal consistency, ICCs, and correlations for all four CAHAI-G versions

n = 23	CAHAI-G 7	CAHAI-G 8	CAHAI-G 9	CAHAI-G 13
Internal consistency	α = 0.979, p ≤ 0.001	α = 0.981, p ≤ 0.001	α = 0.979, p ≤ 0.001	α = 0.974, p ≤ 0.001

ICC	0.967	0.964	0.965	0.989

CI	0.935< × <0.985	0.931< × <0.984	0.932< × <0.984	0.937< × <0.985

CAHAI-G 7	---	r = 0.999	r = 0.994	r = 0.982

CAHAI-G 8	r = 0.999	---	r = 0.997	r = 0.986

CAHAI-G 9	r = 0.994	r = 0.997	---	r = 0.989

CAHAI-G 13	r = 0.982	r = 0.986	r = 0.989	---

α = Cohen's α for internal consistency, CI = confidence interval (95%), ICC = intraclass correlation coefficient, r = correlation coefficient

Inter-item correlations range between r = 0.414 (for 'Clean a pair of eye glasses' and 'Place container on table') and r = 0.945 (for 'Open a jar of coffee' and 'Call 144').

### Convergent construct validity

For convergent construct validity a close relation of CAHAI-G and CMSA subscales arm and hand was hypothesised. The correlation coefficients show a large correlation for CAHAI-G and CMSA subscale hand (r = 0.74) and a moderate correlation for CAHAI-G and CMSA subscale arm (r = 0.67).

## Discussion

By applying the proposed TCCA-OAO procedure for CAHAI a German version of the task instruction manual and scoring descriptions was developed. The eight-stage procedure allowed for a resource-efficient method regarding time and personnel (5 people) involved compared to Beaton et al. (9 people) and Swain-Verdier et al. (6 to 8 people) [3,7,10]. Different from PRO, OAOs benefit from an explicit instruction and scoring description for therapists, which is profession-specific and does not require to be understood by the general population. The control checks of the documents between forward and backward translation by experienced clinicians of the target profession (steps 3 and 6) helped to achieve this goal. With this approach, the particular risk for interpretation differences in OAOs was reduced. A standardised TCCA-OAO is essential for error-free assessment adaptations. With the proposed TCCA procedure five of the six levels of equivalence of Stewart and Napoles-Springer (cited in Acquadro [7]) have been reached: 1) conceptual, 2) semantic, 3) operational, 4) measurement, and 5) item equivalence. The sixth level (criterion equivalence) was not achieved due to the fact that the quality factors were evaluated in a homogeneous patient group.

It is widely accepted that more than one forward translation should be done to create translated versions from different perspectives [1,3,6,7]. However, imposed effort and cost for a TCCA procedure standard must be carefully reviewed. A balance between TCCA quality and invested resources can ensure that standards are considered in clinical practice globally. We are not in line with the suggestion that required back translators should be uninformed native speakers of the original language, living in the target country [3]. Finding adequate translators can require large efforts and raise costs. Moreover, given that the backward translation is validated by the original authors, this requirement appears unnecessary. Thus, we suggest that back translators can be informed native speakers of the target language with excellent language skills of the source language or applying a two-panel approach suggested by Swaine-Verdier et al. [10]. Furthermore, we belief, that the whole TCCA procedure for OAOs is accelerated by having informed forward and backward translators, which was a design choice in this work.

Results of the patient study revealed a very high inter-rater reliability. This was achieved by establishing strict video recording rules during the practical performance of the CAHAI-G: (1) sitting position of the patient and the therapist who was sitting at the affected side of the body and 2) having a second therapist recording the performance from wide-angle and close-up distances in frontal view to emphasise small patient movements. Moreover, the assessor could watch videos as often as necessary.

Our results showed very similar values for inter-rater reliability, validity and internal consistency compared to the original CAHAI which support the suggested TCCA procedure. Larger ICC values obtained for CAHAI-G could be attributed to the video rating performed versus direct performance scoring done in the original. Convergent construct validity could be demonstrated at high to moderate correlations with CMSA subscales for arm and hand.

The small sample size of 23 patients in a subacute or chronic stage of their disease might be a limitation of the current study. Furthermore, we did not sum up CMSA subscale hand and arm scores for the validity calculation, which could have lowered correlation results for CAHAI-G compared to the validation study of the original version. We have found a wide range of inter-item correlations, which we account to the smaller sample size as well.

This study was focussed on the translation, validation and inter-rater reliability evaluation of the CAHAI. Further quality factors, e. g. intra-rater reliability and responsiveness, of the German CAHAI version were not addressed in this work. These evaluations of the CAHAI-G will be the focus of future projects. With the help of the current study the CAHAI-G has been successfully introduced to the occupational department in our rehabilitation centre and will be used in clinical routine.

Further studies are needed to confirm the suggested TCCA procedure in other OAO measures.

## Conclusion

Various approaches and options could be used to translate and culturally adapt OAOs. Our suggested eight step TCCA-OAO procedure is the first of its kind and is based on reviews of existing PRO guidelines and was applied in a practical example that confirmed its applicability. All four versions of the CAHAI-G (CAHAI-G 7, CAHAI-G 8, CAHAI-G 9, and CAHAI-G 13) can be recommended as reliable and valid performance-based measures to assess bilateral upper limb ADL task performance in clinical practice. They can be utilised with patients after stroke in the sub-acute and chronic phase. The shortest version (CAHAI-G 7) needs 10 minutes to perform and the test material is low priced and easy to provide.

We would like to encourage developers of outcome measurements to play an active role in TCCA or even provide official translations of their outcome measure for researchers in foreign countries.

## Abbreviations

**ARAT**: Action research arm test; **CI**: Confidence interval; **CAHAI**: Chedoke Arm and Hand Activity Inventory; **CAHAI-G**: Chedoke Arm and Hand Activity Inventory - German version; **CAHAI-G 7**: Chedoke Arm and Hand Activity Inventory - 7 items to test; **CAHAI-G 8**: Chedoke Arm and Hand Activity Inventory - 8 items to test; **CAHAI-G 9**: Chedoke Arm and Hand Activity Inventory - 9 items to test; **CAHAI-G 13**: Chedoke Arm and Hand Activity Inventory - 13 items to test; **CMSA**: Chedoke-McMaster Stroke Assessment; **ICC**: Intraclass correlation coefficient; **OAO**: Objectively-assessed outcome; **PRO**: Patient-reported outcome measures; **SPSS**: Statistical package for social sciences; **TCCA**: Translation and cross-cultural adaption

## Competing interests

The authors declare that they have no competing interests.

The publication is based on the Master thesis (MSc.) of the second author.

The work has been presented as a poster at the EFNS conference in Florence 2009 [15].

## Authors' contributions

**CSch **has made substantial contribution to conception and design, data analysis and interpretation, and has written the article. **SHa **was involved in the design process. She did the data collection, data analyses and wrote parts of the manuscript. **ThE **contributed to the conception and design of the study. He was involved in the data interpretation process, drafted and critically revised the manuscript. All authors have given final approval of the manuscript.

## Pre-publication history

The pre-publication history for this paper can be accessed here:

http://www.biomedcentral.com/1471-2288/10/106/prepub

## Supplementary Material

Additional file 1 **Appendix: Bewertungsbogen CAHAI-G 13**. German scoring sheet of the CAHAI-G full version (13 items) Click here for file
