# Supplementary material for: Objectively-assessed outcome measures: a translation and cross-cultural adaptation procedure applied to the Chedoke McMaster Arm and Hand Activity Inventory (CAHAI)
Source: BMC Med Res Methodol. 2010 Nov 29;10:106. doi: 10.1186/1471-2288-10-106 (PMC3004924; doi:10.1186/1471-2288-10-106)
Supplement: Additional file 1 — Appendix: Bewertungsbogen CAHAI-G 13. German scoring sheet of the CAHAI-G full version (13 items) [file 1471-2288-10-106-S1.DOC]

##### Appendix: Bewertungsbogen CAHAI-G 13

| **Name:** | **Datum:** |
| --- | --- |

|  | Aktivitätsskala | |  |
| --- | --- | --- | --- |
| **1.** Totale Hilfestellung (FF beeinträchtigte o. E. < 25 %) | | **5.** Supervision | |
| **2.** Maximale Hilfestellung (FF beeinträchtigte o. E . 25 – 49 %) | | **6.** Eingeschränkte Selbständigkeit (Hilfsmittel) | |
| **3.** Mässige Hilfestellung (FF beeinträchtigte o. E. 50 – 75 %) | | **7**. Völlige Selbständigkeit (zeitgerecht, sicher) | |
| **4.** Minimale Hilfestellung (FF beeinträchtigte o. E. > 75 %) | |  | |

FF beeinträchtigte o. E. = Funktionsfähigkeit beeinträchtigte obere Extremität

|  |  | **Beeinträchtigte Extremität** | | | | **Punkte** | |
| --- | --- | --- | --- | --- | --- | --- | --- |
| 1. | Kaffeeglas öffnen | □ | Halt Glas | □ | Hält Deckel |  |  |
|  |
|  |  |
| 2. | Nummer 144 wählen | □ | Hält Hörer | □ | Wählt Nummer |  |  |
|  |
|  |  |
| 3. | Linie mit Lineal ziehen | □ | Hält Lineal | □ | Hält Stift |  |  |
|  |
|  |  |
| 4. | Glas Wasser einschenken | □ | Hält Glas | □ | Hält Kanne |  |  |
|  |
|  |  |
| 5. | Waschlappen auswringen |  |  |  |  |  |  |
|  |
|  |  |
| 6. | Fünf Knöpfe schliessen |  |  |  |  |  |  |
|  |
|  |  |
| 7. | Rücken mit Handtuch abreiben | □ | Reicht nach Handtuch | □ | Ergreift Handtuchende |  |  |
|  |
|  |  |
| 8. | Zahnpasta auf Zahnbürste drücken | □ | Hält Tube | □ | Hält Zahnbürste |  |  |
|  |
|  |  |
| 9. | Mittelharte Knetmasse schneiden | □ | Hält Messer | □ | Hält Gabel |  |  |
|  |
|  |  |
| 10. | Reissverschluss schliessen | □ | Hält Reiss-verschluss | □ | Hält Reiss-verschlusssschieber |  |  |
|  |
|  |  |
| 11. | Brille putzen | □ | Hält Brille | □ | Putzt Gläser |  |  |
|  |
|  |  |
| 12. | Kunststoffbehälter auf den Tisch stellen |  |  |  |  |  |  |
|  |
|  |  |
| 13. | Tasche eine Treppe hinauftragen |  |  |  |  |  |  |
|  |
|  |  |
|  |  | **Gesamtpunktezahl** | | | |  | **/91** |
|  |  |

| Anmerkungen |
| --- |
|  |

*CAHAI-G: deutsche Übersetzung des Chedoke-McMaster Arm and Hand Activity Inventory (CAHAI); Barreca SR et al. Test-Retest Reliability, Validity, and Sensitivity of the Chedoke Arm and Hand Activity Inventory: A New Measure of Upper-Limb Function for Survivors of Stroke. Arch Phys Med Rehabil Vol 86, August 2005

1/1
